# Basal Ti level in the human placenta and meconium and evidence of a materno-foetal transfer of food-grade TiO_2_ nanoparticles in an ex vivo placental perfusion model

**DOI:** 10.1186/s12989-020-00381-z

**Published:** 2020-10-07

**Authors:** A. Guillard, E. Gaultier, C. Cartier, L. Devoille, J. Noireaux, L. Chevalier, M. Morin, F. Grandin, M. Z. Lacroix, C. Coméra, A. Cazanave, A. de Place, V. Gayrard, V. Bach, K. Chardon, N. Bekhti, K. Adel-Patient, C. Vayssière, P. Fisicaro, N. Feltin, F. de la Farge, N. Picard-Hagen, B. Lamas, E. Houdeau

**Affiliations:** 1 Toxalim UMR1331 (Research Centre in Food Toxicology), Toulouse University, INRAE, ENVT, INP-Purpan, UPS, Toulouse, France; 2 Department of materials, LNE, Trappes, France; 3 Department for biomedical and inorganic chemistry, LNE, Paris, France; 4 Group Physic of Materials, GPM-UMR6634, CNRS, Rouen University, Rouen, France; 5 Department of Obstetrics and Gynecology, Paule de Viguier Hospital, CHU Toulouse, Toulouse, France; 6 INTHERES, UMR 1436 Toulouse University, INRAE, ENVT, Toulouse, France; 7 Péritox UMR-I 01 (Perinatality and Toxic Risk), Jules Verne University, Amiens, France; 8 Université Paris Saclay, CEA, INRAE, Département Médicaments et Technologies pour la Santé (DMTS), SPI, 91191 Gif-sur-Yvette, France; 9 UMR 1027 INSERM, Team SPHERE, Toulouse III University, Toulouse, France

**Keywords:** Titanium dioxide, Nanoparticles, Human placenta, E171 food additive, Foetus

## Abstract

**Background:**

Titanium dioxide (TiO_2_) is broadly used in common consumer goods, including as a food additive (E171 in Europe) for colouring and opacifying properties. The E171 additive contains TiO_2_ nanoparticles (NPs), part of them being absorbed in the intestine and accumulated in several systemic organs. Exposure to TiO_2_-NPs in rodents during pregnancy resulted in alteration of placental functions and a materno-foetal transfer of NPs, both with toxic effects on the foetus. However, no human data are available for pregnant women exposed to food-grade TiO_2_-NPs and their potential transfer to the foetus. In this study, human placentae collected at term from normal pregnancies and meconium (the first stool of newborns) from unpaired mothers/children were analysed using inductively coupled plasma mass spectrometry (ICP-MS) and scanning transmission electron microscopy (STEM) coupled to energy-dispersive X-ray (EDX) spectroscopy for their titanium (Ti) contents and for analysis of TiO_2_ particle deposition, respectively. Using an ex vivo placenta perfusion model, we also assessed the transplacental passage of food-grade TiO_2_ particles.

**Results:**

By ICP-MS analysis, we evidenced the presence of Ti in all placentae (basal level ranging from 0.01 to 0.48 mg/kg of tissue) and in 50% of the meconium samples (0.02–1.50 mg/kg), suggesting a materno-foetal passage of Ti. STEM-EDX observation of the placental tissues confirmed the presence of TiO_2_-NPs in addition to iron (Fe), tin (Sn), aluminium (Al) and silicon (Si) as mixed or isolated particle deposits. TiO_2_ particles, as well as Si, Al, Fe and zinc (Zn) particles were also recovered in the meconium. In placenta perfusion experiments, confocal imaging and SEM-EDX analysis of foetal exudate confirmed a low transfer of food-grade TiO_2_ particles to the foetal side, which was barely quantifiable by ICP-MS. Diameter measurements showed that 70 to 100% of the TiO_2_ particles recovered in the foetal exudate were nanosized.

**Conclusions:**

Altogether, these results show a materno-foetal transfer of TiO_2_ particles during pregnancy, with food-grade TiO_2_ as a potential source for foetal exposure to NPs. These data emphasize the need for risk assessment of chronic exposure to TiO_2_-NPs during pregnancy.

## Introduction

Titanium dioxide (TiO_2_) is one of the most commonly produced nanomaterials used for various industrial applications (cosmetics, water and soil treatment, UV filter, medicine, food sector) based on its colouring and opacifying properties as well as its photocatalytic and biocidal activities [1, 2]. Due to these widespread applications, including in food, human exposure to TiO_2_ nanoparticles (NPs, at least one dimension < 100 nm) occurs by inhalation, dermal exposure and the oral route. Translocation across biological barriers (lung, skin, intestine) depends on the crystal form (anatase or rutile), coating, size, surface area and aggregate/agglomerate formation [3]. A systemic passage for TiO_2_ is documented in human [4–6] and resembles that reported in rodents [7, 8], with accumulation in the liver and spleen of nano- and submicronic particles [9], indicating a low but chronic distribution of TiO_2_ particulate matter in the human organism**.** In vivo studies in rodents exposed to TiO_2_ reported toxic effects such as inflammation, impairment of biological barrier functions (intestinal, placental, blood-testis), as well as the promotion of cancer development [7, 10, 11]. Overall, repeated exposure to these particles exhibiting cytotoxic, genotoxic and immunotoxic effects is considered to be an important health issue [12–14]. For the general population, there is accumulating evidence that the main uptake route is ingestion, since TiO_2_ is produced at high volumes as a food grade pigment (E171 in EU) incorporated in many foodstuffs as well as in toothpaste, medicines and food supplements. Dietary exposure to TiO_2_ ranges from 0.4 to 10.4 mg/kg BW/day [15], depending on the age group and food habits. E171 is a powder consisting of anatase and/or rutile TiO_2_, exhibiting up to 55% NPs by number depending on the commercial supplier [7, 16]. Oral kinetic studies in human volunteers showed that a fraction of food-grade TiO_2_ is absorbed and reaches the bloodstream a few hours after ingestion [5]. However, in this context, maternal exposure during pregnancy has not yet been evaluated for risk assessment in humans. Chronic oral intake of TiO_2_-NPs in pregnant women could lead to placental dysfunction and/or transplacental passage, both of which may have deleterious impacts on foetal development [11, 17, 18].

Due to the separation of maternal and foetal circulations, the placenta acts as a barrier to environmental toxicants [19, 20] and potential pathogens [21] that could be hazardous for the growing foetus. This barrier was nevertheless shown to be poorly effective when exposed to TiO_2_-NPs, at least in mice and rats, with evidence of particle accumulation in the placenta and passage to the foetus [22]. Toxicity data indicate resorption of embryos and foetal growth restriction in mice exposed intravenously to TiO_2_-NPs for two days at mid-pregnancy [17]. Moreover, low doses of TiO_2_-NPs orally administered to dams during the first two gestational weeks were shown to impair placentation and induced dysregulation of vascularization and proliferation, possibly leading to alteration of nutrient and gas exchanges with the foetus [11]. Other studies in rodents exposed through oral route [23–25] or intravenous injection [17, 26] throughout gestation showed a materno-foetal transfer of TiO_2_-NPs and their accumulation in several foetal organs, such as the liver, testis and brain, the latter distribution leading to an impairment of cognitive functions in the offspring. However, all these hazards cannot be directly transposed to humans for risk assessment due to interspecies differences in placentation and structure [27, 28]. Recently, one study reported a significant titanium (Ti) content in the term placentae and in the cord blood in paired mothers-infants, suggesting that a placental transfer of TiO_2_ may also exists in humans [29]. Nevertheless, specific data concerning the human placental and meconium content of TiO_2_ particles (and particularly NPs) and their transfer are currently lacking. The meconium could be the best matrix to analyse in utero exposure to TiO_2,_ as it is the first neonatal stool accumulated during the last 6 months of pregnancy in the foetal intestine. Analysis of meconium, which is naturally excreted within the first two days of life and usually discarded, is non-invasive and considered highly accurate to detect chronic foetal exposure to xenobiotics [30].

In humans, studies on the transplacental passage of inorganic particles have been conducted in vitro on trophoblastic cells and ex vivo using isolated perfused placenta. The size was reported an important contributing factor for particle transfer. Ex vivo, only polystyrene beads up to 240 nm crossed the syncytiotrophoblast that separates the foetal circulation from the maternal blood [31], with low transfer rates and bidirectional passage (i.e., materno-foetal and inversely) [32]. In vitro, 25 and 50 nm silicon (Si) NPs were reported to cross the placental barrier, an observation confirmed ex vivo using perfused human placenta [33]. These findings suggest the capacity for the nanosized fraction (< 100 nm) of food-grade TiO_2_ to reach the foetus. To date, in the absence of specific information on food-grade formulations, two studies have examined the capacity of TiO_2_-NP models perfused with different surface charges to cross human placenta. These studies failed to observe a quantifiable increase in Ti levels in the foetal compartment using inductively coupled plasma mass spectrometry (ICP-MS) analysis after 6 h of perfusion, but authors do not exclude the possibility that a low amount of NPs may cross the placental barrier in humans [34, 35], which remains highly challenging to detect. Even in the case of low placental transfer rates, foetal accumulation of insoluble TiO_2_-NPs may occur if the foetus is chronically exposed via its mother to TiO_2_ of various environmental origins, including dietary sources.

In this study, we aimed to assess TiO_2_ exposure in the human foetus by performing ICP-MS analysis of Ti contents in the human placenta collected at term from pregnancies and in the meconium. These matrices were also analysed with scanning transmission electron microscopy coupled to energy dispersive X-ray (STEM-EDX) analysis to ensure the presence of particles and their chemical nature. Second, by using the ex vivo human placenta perfusion model, we assessed whether TiO_2_-NPs of dietary origin (E171 additive) may cross the placental barrier by combining ICP-MS, confocal microscopy, scanning electron microscopy (SEM) and STEM-EDX for compositional analysis and elemental mapping of the perfused samples in the foetal side and particle distribution into perfused placental tissues.

## Results

### Physico-chemical characteristics of food-grade TiO_2_ particles

**Fig. 1:**
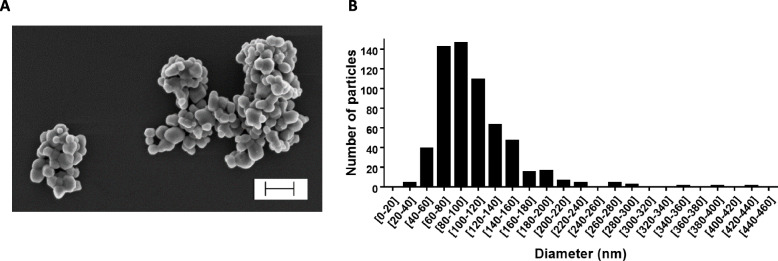
SEM analysis of size distribution of food-grade TiO_2_ particles. **a** Scanning electron microscopy image of TiO_2_ particles in E171 water suspension. Scale bar = 300 nm. **b** Primary size distribution by number of TiO_2_ particles, based on the diameters of 600 particles measured on SEM images

SEM-EDX analyses showed that E171 had TiO_2_ particles with a mean particle size of 104.9 ± 44.9 nm and a particle size distribution ranging from 20 to 440 nm, with 55% of NPs by number (Table 1 and Fig. 1). The specific surface area (SSA) was shown to be 9.6 m^2^/g by BET, and the zeta potential in water suspension was − 35.1 ± 3.22 mV (Table 1). DLS analysis showed increased hydrodynamic diameters of the TiO_2_ particles in Earle’s medium (Table 1), indicating particle agglomeration in the perfusion medium (PM) compared to ultrapure water.

**Table 1:** Physico-chemical characteristics of E171

			Ultrapure water (pH = 8.92)			Perfusion medium (pH = 7.40)		
Mean particle size (nm)	% of NPs	SSA (m^**2**^/g)	Zeta potential (mV)	H. diam. (nm)	PdI	Zeta potential (mV)	H. diam. (nm)	PdI
104.9 ± 44.9	55	9.6	−35.1 ± 3.22	303.27 ± 134.27	0.223	−12.3 ± 0.00	383.3 ± 163.6	0.363

All data are presented as mean ± SD. *SSA* specific surface area, *H. diam*. hydrodynamic diameter, *PdI* polydispersity index

### ICP-MS measurement of total Ti content in the human term placenta and the meconium, and particle analysis by STEM-EDX

**Fig. 2:**
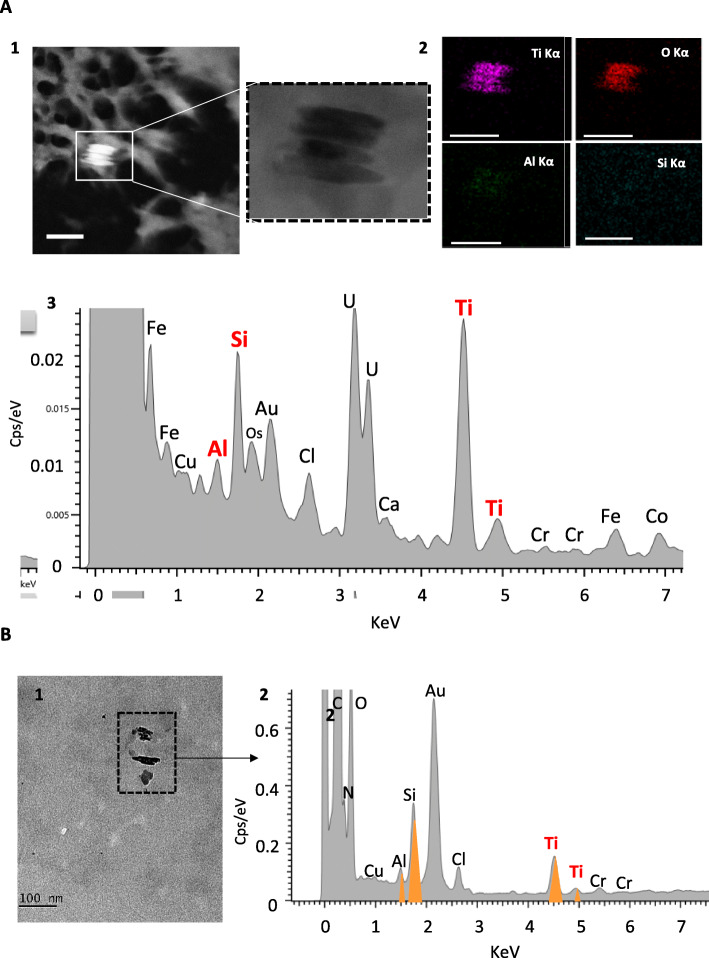
STEM-EDX analysis of TiO_2_ particles basal distribution in human placenta (**a**) and meconium (**b**). (A1) (S) TEM -micrographs showing cluster of four particles. Left hand corner micrograph is acquired in STEM-HAADF mode where particles appear brighter than biological matrix, due to the detection of elastic electron scattered by high Z number particles (chemical contrast). (A2) STEM-EDX elemental maps and sum spectrum identifying Ti, O, Al, and Si as main elements, and (A3) corresponding EDX analysis. All scale bars = 100 nm. (B1) TEM image illustrating particle content in meconium and coupled to EDX analysis (B2) with Ti, O, Al and Si identified as main elements over 3 particles

As shown in Table 2, Ti was found in all placental samples (*n* = 22), with the total Ti content ranging from 0.01 to 0.48 mg/kg of tissue. Seven women displayed a total placental Ti content above 0.1 mg/kg of tissue, with 2 of them reaching 0.4–0.5 mg/kg of tissue. On TEM tissue sections, particulate matter was observed as isolated primary particles and/or as small aggregates in placental tissues. Chemical elemental mapping using STEM-EDX confirmed the presence of Ti and oxygen (O) onto particle deposits (Fig. 2a), often appearing as elongated crystal forms associated with aluminium (Al) and Si trace elements, and most of the analysed TiO_2_ particles were below 100 nm in diameter (Fig. 2a). Among the 34 particles analysed by EDX, Si-, tin (Sn)-, Al-, iron (Fe)- and zinc (Zn)-containing particles were frequently found in addition to TiO_2_ (Fig. S1). Size measurements of all particles (i.e., whatever their chemical nature) recovered during the TEM analysis showed that 32 of 34 particles exhibited diameters ranging from 10 to 225 nm, 16 of them (i.e., 50%) being below 100 nm (Fig. S2), and two large agglomerates of 320 and 380 nm (Fig. S2).

**Table 2:** ICP-MS analysis of basal Ti content in human term placenta and meconium (unpaired samples)

	Total Ti (mg/kg)		
	Placenta (***n*** = 22)		Meconium (***n*** = 18)
**1**	0.04	**1**	0.19
**2**	0.02	**2**	0.09
**3**	0.01	**3**	*< LOQ*
**4**	0.05	**4**	0.17
**5**	0.20	**5**	*< LOQ*
**6**	0.15	**6**	*< LOQ*
**7**	0.03	**7**	0.34*
**8**	0.17	**8**	1.41*
**9**	0.11	**9**	*< LOQ*
**10**	0.05	**10**	*< LOQ*
**11**	0.03	**11**	0.02
**12**	0.01	**12**	*< LOQ*
**13**	0.16	**13**	*< LOQ*
**14**	0.02	**14**	0.25*
**15**	0.47	**15**	*< LOQ*
**16**	0.06	**16**	0.28*
**17**	0.09	**17**	1.50*
**18**	0.04	**18**	*< LOQ*
**19**	0.08		
**20**	0.48		
**21**	0.01		
**22**	0.03		
**n > LOQ**	100%		50%
**average**	0.10		0.47 0.57
**SD**	0.13		
**min**	0.01		0.02
**max**	0.48		1.50
**median**	0.05		0.25
**1st quartile**	0.03		0.17
**3rd quartile**	0.14		0.34

Samples were obtained from unpaired mother-infants. All concentrations are corrected for total blank signals. *Weighted mean for 3 analyses. LOQ = 0.01 mg/kg for meconium and 0.003 mg/kg for placenta. Typical relative measurement uncertainties were 6.5% (k = 1) for meconium, and 8% (k = 1) for placenta

In the meconium (Table 2), Ti was detected by ICP-MS in 50% of samples (9 of 18), ranging from 0.02 to 1.50 mg/kg of material. In most of these samples (*n* = 8), the Ti levels were above 0.1 mg/kg of meconium (Table 2). TEM-EDX analysis of two representative meconium samples confirmed the presence of Ti and O elements in the particulate deposits (Fig. 2b). In addition, clustered elements of mainly Si, Al, Fe and Zn were observed as particulate matter (Fig. S3). Overall, size analysis of all particles indicated a diameter ranging from 5 to 194 nm (*n* = 33 particles), and 26 of them (i.e., 82%) were in the nanorange.

### Transfer profile of food-grade TiO_2_ particles across the human placenta ex vivo

**Fig. 3:**
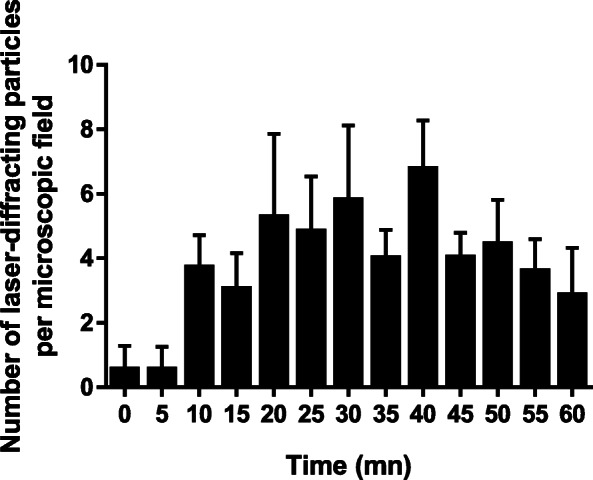
Mean transfer profile of particles during 1 h of placental perfusion with E171 using confocal microscopy. Placenta were perfused with food-grade TiO_2_ (E171, 15 μg/mL in PM) and counted as laser-diffracting particle spots over 1 h of experiment. Each data is the mean ± SEM of 4 to 6 independent experiments

A total of 7 placentae were validated based on antipyrine passage during ex vivo perfusion in an open circulating system with PM alone and E171 suspension. There was no difference in permeability (i.e., antipyrine transfer rate) between placentae collected after caesarean section or vaginal delivery (Fig. S4). After the perfusion of placentae with food-grade TiO_2_ (E171), Ti was detected by ICP-MS in most foetal exudates (0.41 to 3.46 ng Ti/mL), but 92% were in the range of the blank level (0.33 to 1.92 ng Ti/mL) (Table S1). To assess whether a transfer occurred for TiO_2_ particles, we examined the exudate samples collected every 5 min on the foetal side by confocal microscopy to detect laser-diffracting (TiO_2_-like) particles, as shown in Fig. S5. An average blank value of 1.05 ± 0.32 laser-diffracting spots per microscopic field was calculated from 4 independent experiments with PM free of E171, and then subtracted from each time point in each placenta perfused with the E171 suspension. As shown in Fig. 3, the placental translocation of laser-diffracting TiO_2_ particles increased from 10 min of E171 perfusion, reached a plateau at 20–30 min, and then decreased until the end of the perfusion.

### SEM-EDX analysis of perfused particles recovered in the foetal exudate

**Fig. 4:**
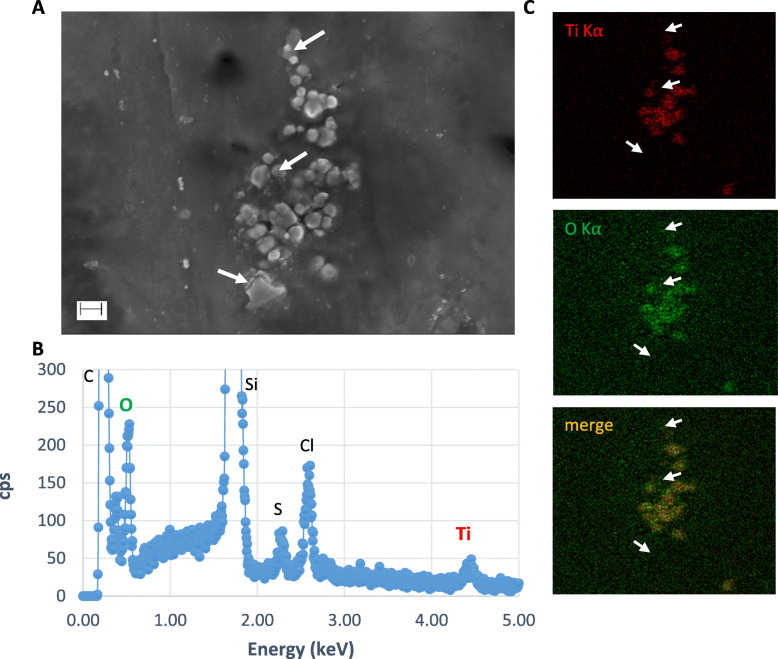
Analysis of TiO_2_ particles by SEM-EDX in foetal exudate. **a** SEM images of particles agglomerated in the dried PM after sample preparation of foetal exudate taken up from the 20–30 min period of perfusion. **b** Corresponding EDX analyses of particles into agglomerates showing the presence of C, Cl, S, Ti and O (Si signal from SEM wafer). **c** SEM-EDX cartography of particles showing Ti (red) and O (green) elements, and corresponding merge image. White arrows indicate examples of particles negative for Ti and O in this preparation. Scale bar = 200 nm

**Fig. 5:**
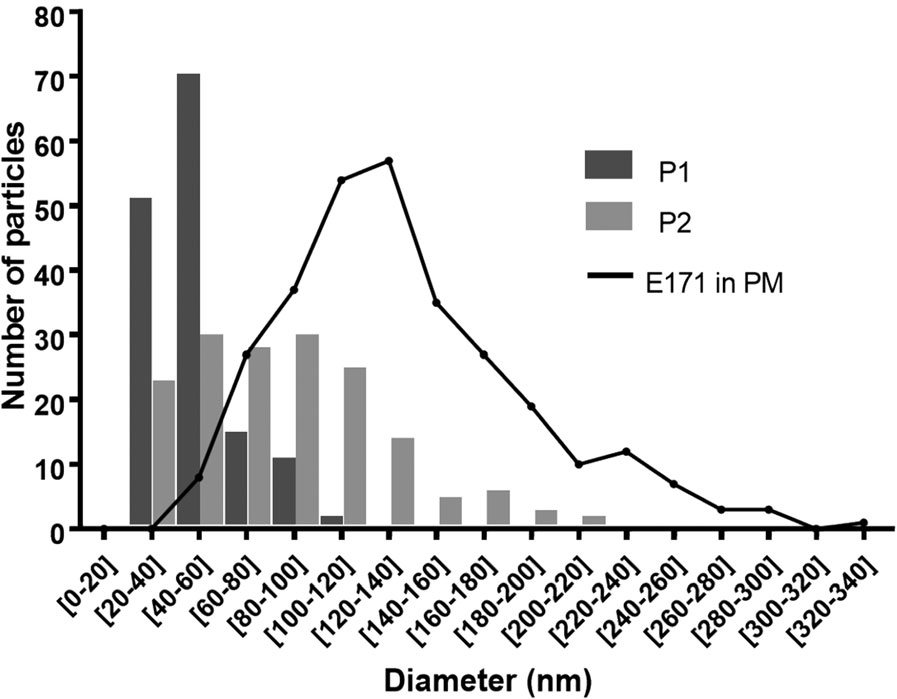
Size distribution of TiO_2_ particles in the maternal perfusion medium and the foetal exudate. Primary size distribution of TiO_2_ particles by number based on diameters measured on SEM-EDX images corresponding to samples of foetal exudate collected between 20 and 30 mn of E171 perfusion in 2 placentae, and compared to E171 suspension (solid line) in the perfusion medium (PM). P1 = 144 particles, P2 = 156 particles, and E171 in PM = 300 particles

Based on the above reported particle transfer profile with confocal imaging, pooled liquid fractions in the foetal side corresponding to the 20–30 min period of perfusion with the E171 suspension (see Fig. 3) were subjected to SEM-EDX analysis. After sample purification, SEM-EDX scanning showed the presence of many TiO_2_ particles appearing as isolated particles or aggregates trapped in the matrix of the dried PM (Fig. 4). Furthermore, Al and Fe elements were also found in some particulate deposits, associated or not with Ti (Fig. S6). The current detection method by SEM-EDX did not provide quantitative information on the total number of TiO_2_ particles recovered in the foetal circuit over the 20–30 min of E171 perfusion but allowed us to evaluate the size distribution of the particles crossing the placenta during this period. As a result, a total of 300 TiO_2_ particles sampled and purified from the foetal side were measured by taking the smallest dimension observed for each TiO_2_-positive particle in the recovered agglomerates (Fig. S7), and the results compared to the TiO_2_ particle size distribution in the PM with E171 added from the maternal reservoir (Fig. 5). Except for 3 particles, TiO_2_ particles recovered in the foetal side exhibited a diameter < 200 nm, with 70 and 100% of NPs in two perfusion experiments (Fig. 5).

### STEM-EDX analyses of TiO_2_ particles in the perfused placental tissues

**Fig. 6:**
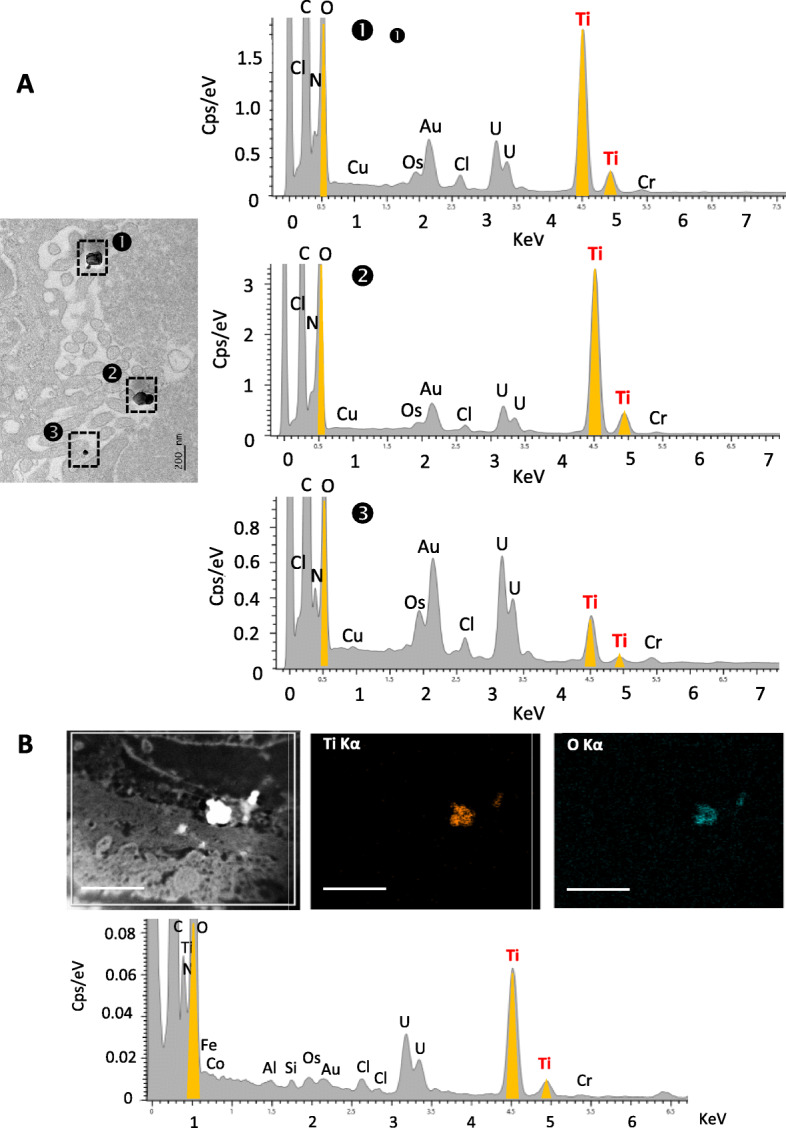
Tissue distribution of TiO_2_ particles in the E171 perfused placenta. **a** TEM-Bright-Field micrographs coupled to EDX analysis showing food-grade TiO_2_ particles into the syncytiotrophoblast microvilli. **b** Chemical characterization of TiO_2_ particles by STEM-EDX mapping based on STEM-HAADF micrographs for particle detection, and elemental maps and sum spectrum. Scale bars in (B) = 1 μm

In placental tissue, (S)TEM-EDX was used to investigate the distribution of TiO_2_ particles into the perfused cotyledon. As illustrated in Fig. 6, enriched tissue areas with Ti + O showed isolated round shaped particles or small aggregates of TiO_2_, typical of the food-grade batch used in this study. TiO_2_ particles from the E171 suspension were recovered in the syncytiotrophoblast microvilli (Fig. 6a and S8D) and had translocated in deeper areas of the placental chorionic mesenchyme surrounding foetal vessels (Fig. 6b and S8D). As shown in Fig. S8, size measurement of the 33 TiO_2_ (i.e., EDX-characterized) particles translocated into placental tissues showed 26 particles with a diameter below 250 nm, and 17 of them in the nanorange.

## Discussion

The effects of prenatal exposure to xenobiotics (potentially toxic) on birth outcomes and child development are an area of concern for public health [36], which have been extended to NPs of anthropogenic origin, including TiO_2_-NPs. For pregnant women, a recent opinion by the French High Council for Public Health [37] indicated that the risk of a transfer of TiO_2_-NPs to the foetus and the health consequences for the newborn have not been documented despite animal studies showing harmful effects [22], with TiO_2_-NPs recovered in foetal organs such as the brain, liver and testis [17, 23, 26]. A recent study in humans analysed the Ti content in maternal and cord blood and suggested a high placental transfer efficiency of Ti [38]. Titanium was also evidenced in the amniotic fluid, even if the prevalence of pregnant women exhibiting amniotic Ti load is quite low [39]. However, whether Ti signal originated from (nano) particulate matter was not specified and, to date, no analytical data exist on the potential accumulation of TiO_2_ particles in the human placenta and the meconium, the latter being used herein as a biomarker of prenatal exposure of the newborn to TiO_2_ from the mother. In this study, a combination of two analytic methods with ICP-MS and STEM-EDX allowed us to determine the total Ti content in these biological matrices together with the morphology, size and chemical characterization of the particles identified in the placental tissue sections and in the meconium preparations for electron microscopy. We report that all collected placentae from term pregnancies had a quantifiable amount of Ti, with an average level of 0.10 mg/kg of tissue, which is consistent with the findings of Li et al. [29] (0.2 mg/kg) and close to the Ti concentrations under particulate form in the human liver and spleen, as previously measured by single particle (sp) ICP-HRMS in organs recovered post-mortem [9]. Furthermore, seven placentae exhibited Ti contents between 0.1 and 0.5 mg/kg of tissue, these amounts being comparable to the maximum Ti levels reported in the human spleen [9], suggesting a high capacity of the placenta to accumulate Ti(O_2_) from the maternal blood. TEM imaging coupled to EDX analysis showed the presence of isolated and clustered TiO_2_ particles; most of the constitutive particles were spherical and assumed to have an anatase crystal structure, while others consisted of elongated TiO_2_ particles typical of the rutile forms. The rutile and anatase forms of TiO_2_ are authorized in the formulations of the E171 food additive in the EU (EC Regulation n°231/2012), while rutile is also commonly used in sunscreens and cosmetics such as day creams and loose and pressed powders [40, 41], with possible inhalation and/or absorption through the skin, although very few studies have revealed dermal penetration through intact skin [3]. Furthermore, TiO_2_ is used as an opacifying agent in indoor paints with particles released into domestic air with dust and pulmonary exposure [42–44]. However, all these different routes of exposure cannot be discriminated on the sole base of the crystal forms recovered in the tissue due to their different usages both in the environment and in consumer products. Furthermore, we consistently identified particle deposits of Al, Si, Fe, Sn and Zn in the term placentae. These accumulated particles may originate from dietary intake [45] and pharmaceutical products [5] along with other sources such as air pollution in an urban environment [46–48]. With the example of tin, our data showing deposition as particles in the human placenta are consistent with the exposure profiles to Sn measured in the maternal blood and the tin accumulation in the placenta as reported by ICP-MS measurements [49], which requires further research regarding placental transfer.

For meconium, 50 % of the samples had a quantifiable amount of Ti (range 0.02–1.50 mg/kg), with some of them exhibiting Ti contents well above those recovered in the placenta. Meconium reflects a long window of exposure and is considered a relevant matrix in toxicology screening for detecting foetal exposure to various xenobiotics, such as airway pollutants, heavy metals, pesticides or pharmaceuticals, with which pregnant women are in contact [30, 50]. These chemicals can migrate into the foetus directly from the placental interface through the cord blood or come from the amniotic fluid of which formation partly results from transudation of maternal fluids together with secretions of the amnion epithelium [51]. Because meconium matrix contains amniotic fluid mixed with foetal urines, all being continuously swallowed (as well as inhaled) by the foetus during pregnancy, and reabsorbed by the foetal intestine [51], such matrix may reflect global foetal body exposure to TiO_2_. From the current ICP-MS analysis, the high variability of Ti content among the meconium samples, as shown in placenta, suggests inter-individual differences in mothers’ exposure to TiO_2_ and/or in basal permeability of biological barriers to TiO_2_ that remain unexplored. Given the maternal origin of xenobiotics in meconium [30], it is assumed that foetal exposure to TiO_2_ directly relies on the mother’s use of TiO_2_-containing products and/or TiO_2_ emission in her environment, which may have multiple origins on a household basis, as noted above. In our study, further EDX analysis confirmed TiO_2_ particle deposition in the meconium. Furthermore, all the recovered TiO_2_ particles were nanosized and often exhibited morphology resembling that of elongated TiO_2_ (putative rutile) structures, as observed in the placental tissues. In addition, Fe particles and particulates deposits with Al and Si were found in the meconium as in the placentae. Notably, materno-foetal translocation of Si-NPs has been demonstrated in vitro in the placental BeWo cell line [33], which represents the rate-limiting barrier for maternal-foetal exchange, as well as ex vivo in perfused human placenta [33] and in vivo in mice [52]. For Al and Si particle deposits, as noted above, both elements are ubiquitous in the environment and diet [45, 53, 54]. Finally, for TiO_2_, because the present study was not conducted on mother-child pairs, it is not possible to relate the Ti levels measured in the meconium to the amount found in the placenta. Nonetheless, altogether, the current findings provide evidence of a materno-foetal passage of TiO_2_ nanoparticles in humans.

To confirm this passage, we conducted a study using the ex vivo human placenta perfusion model considered the gold standard for studying human materno-foetal transfer of xenobiotics [55], including nanoparticles [31, 32]. We focused on E171 food additive due to recent risk assessment studies highlighting oral ingestion as the major TiO_2_ route of exposure in the general population [9, 56]. This conclusion was based on the substantial daily ingestion of food-grade TiO_2_ used as an E171 additive (up to several milligrams per kilogram of BW per day [15]) in the absence of an acceptable daily intake, and on toxicokinetic information for accumulation in organs and clearance from tissues [56]. We first showed poorly detectable Ti signals in the foetal exudate, suggesting a passage of particles too low to be distinguished from the blank levels after 1 h of E171 perfusion. Similar observations have been reported by other groups after 6 h of perfusion with TiO_2_-NPs models, with Ti signals in the range of the background levels of the control perfusion medium [34, 35], as herein reported. In these studies, including the present, the TiO_2_ concentration used for perfusion (10 to 25 μg/mL for toxicokinetic purposes) was approximately 1000 times higher than the basal Ti blood level in humans (10 μg/L) [4, 5] in order to optimize particle detection under a short time of perfusion (i.e., maximum 6 h) and given the short viability of the placenta ex vivo in a non-recirculating system. A high TiO_2_ concentration in perfusion medium could rapidly lead to clogging of the intervillous space on the maternal side, limiting particle recovery on the foetal side. Indeed, in the study of Wick et al. (2010) using 25 μg/mL PS beads for a 6 h perfusion, nanosized beads were reported to cross the placental barrier only during the first hour, with no subsequent transfer when the PS beads were de novo added after 3 h in the maternal compartment. The authors suggested that the high dose of particles in the intervillous spaces created an agglomeration close to the placental villus on the maternal side, preventing the transfer of beads to the foetal compartment after a certain time [31]. These results suggested that a similar situation for TiO_2_ particles could explain the low Ti levels on the foetal side, as reported in the above perfusion studies [34, 35]. In our study, we focused on a one-hour perfusion to limit the risk of placental obstruction by TiO_2_ particles from the perfusion medium with E171.

To avoid uncertainties in our study, we used confocal microscopy for particle visualization in the foetal effluent (i.e., appearing as laser-diffracting metal particles in the exudate) and SEM-EDX analysis to ascertain their chemical nature as TiO_2_ particulate matter. Based on the confocal transfer profile, a progressive increase of the TiO_2_ particles was recovered in the foetal side as soon as 10 min after the E171 suspension was added in the maternal compartment. Interestingly, a rapid passage was also reported for gold (Au) NPs (20 nm) across the rat placenta via ex vivo perfusion, i.e., within 20 min following material infusion [57]. In our study, given the poorly quantifiable level of Ti by ICP-MS in the foetal effluent following E171 perfusion, it is assumed that the mean transfer rate of the TiO_2_ particles over time is very low. However, given that our experiment was conducted for only 1 h, the overall transfer of particles during the whole duration of pregnancy could account for a non-negligible accumulation of TiO_2_ (nano) particles in the foetal body. This assumption is well supported by our above demonstration of the presence of Ti in 50% of the meconium samples collected for this study. Furthermore, we showed that a large majority of the TiO_2_ particles found in the foetal exudate after E171 perfusion was in the nanorange. Because the size distribution of TiO_2_ particles recovered into tissues of a perfused cotyledon showed 50% composed of TiO_2_ nanoforms, this suggested that the fraction of larger TiO_2_ particles remained mostly trapped into the placental tissues and did not reach the foetal compartment. Both these findings indicate that the placenta cannot be considered as an absolute barrier preventing the passage of TiO_2_ NPs from the maternal blood, as previously reported using PS beads or Au-NPs with similar perfusion approaches [31, 58]. To date, the mechanisms for NP translocation are still unknown, but the main hypothesis for NP transport involves transtrophoblastic channels and/or endocytotic mechanisms across the placental barrier [32, 58, 59].

From a risk perspective, animal studies raised concerns about NPs entering the foetus in rodents exposed through inhalation or the transcutaneous or oral route. A large variety of effects on developmental processes have been reported for TiO_2_-NPs, particularly on brain functions due to translocation through the foetal blood–brain barrier, with consequences on behaviour [22]**.** In addition, because immunotoxic and antibacterial properties have been reported for TiO_2_ (nano) particles, including the food-grade form [7, 60–63], their accumulation in the foetal gut through the meconium, as shown herein, could affect the primary colonization of the intestine by the microbiota at birth as well as the maturation of the intestinal immune system, two perinatal events of which dysfunctions have long-term health consequences, as recently reviewed [64]. Accumulation in the placenta may also lead to placental dysfunction (e.g., dysregulation of vascularization, inhibition of cell proliferation, induction of apoptosis) and subsequent foetal growth restriction [11]. In addition to the findings in these rodent studies, TiO_2_-NPs can trigger autophagy and mitochondrial dysfunction in vitro in human trophoblast cells [65, 66], and further impair the cell migration [67] that is needed for trophoblastic invasion in the uterine wall, permitting embryo implantation. Therefore, additional studies are needed to quantify and further characterize the human foetal exposure to TiO_2_-NPs shown in our study, together with studies in animals chronically exposed to TiO_2_ during pregnancy, including from the oral route, to determine the potential hazards for foetal development and newborn health.

## Conclusion

By combining different methods for particle detection, element composition and size analysis together with Ti quantification, the present study highlighted the passage of TiO_2_ particles across the human placenta with potential local accumulation during pregnancy depending on individuals. Even if the placental transfer could not be quantified, a translocation to the foetal body seems to exist, as reflected by the Ti content and TiO_2_ particle deposits in meconium samples, mainly of nanosized forms, that suggest prenatal TiO_2_ exposure from various sources. We further demonstrated TiO_2_ particle transfer through isolated human placenta perfused with food-grade TiO_2_ from the E171 additive, concluding that the human placental barrier is unable to completely prevent the passage of TiO_2_-NPs from dietary sources and protect the foetus. Finally, given the current study showing ex vivo a materno-foetal passage of nanosized TiO_2_ particles in humans, together with quantitative data from different cohorts showing placental and meconium Ti load at term of pregnancy, our findings emphasize the need to assess the risk of TiO_2_-NP exposure in pregnant women and warrant specific attention for oral exposure to the nanosized fraction of the E171 food additive.

## Methods

### Particle characterization and preparation

The E171 food additive was purchased as powder from the website of a French commercial supplier of food colouring agents. These food-grade TiO_2_ particles were prepared following the generic Nanogenotox dispersion protocol as previously described [7]. The E171 stock suspension (25.6 mg/mL**)** was sonicated in an ice bath for 16 min at 30% amplitude (VCX 750-230 V, Sonics Materials) to obtain a stable dispersion of TiO_2_ particles and then stocked at 4 °C during 15 days maximum before use. Dynamic light scattering (DLS, ZetaSizer nano ZS; Malvern Instruments Ltd.) measurements were performed on TiO_2_ particles in ultrapure water (pH = 8.92) and perfusion medium (PM = Earle + 2% BSA, pH 7.4). Five μL of E171 stock suspension were diluted in 3 mL of PM and the hydrodynamic diameter (Z-average), polydispersity index and zeta potential of the TiO_2_ particles were measured. Particle diameters were measured by scanning electron microscopy (SEM**,** *n* = 600 particles) and are expressed as the percentage of NPs by number. The specific surface area (SSA) of the particles was assessed according to the Brunauer, Emmet and Teller method (BET).

### Human placenta and meconium collection

The study was conducted in accordance with the Declaration of Helsinki and its later amendments. Placentae and meconium were collected from different sites and thus were not from mother-infant pairs. Term placentae were collected from 22 uncomplicated pregnancies in the CHU Paule de Viguier (Toulouse, France; Institutional approval (DC-2013-1950). Signed informed consent was obtained from all the mothers. Meconium samples (< 48 h post-partum) were collected in the maternity ward of Sainte-Thérèse Clinic (Paris, France), under an internal agreement of the scientific committee (*n* = 11), or from volunteer parents (*n* = 7). Informed signed consents were obtained from all parents. Meconium collection is non-invasive as it is directly recovered by scraping stained diaper with a sterile disposable spatula, taking care not touching nappy surface.

For basal Ti level determination in all placentae and meconium, a biopsy from one cotyledon per placenta and all meconium samples were weighed and stored at − 80 °C before analysis. Samples from 2 placentae and 2 meconium were also prepared for STEM-EDX analysis.

For ex vivo perfusion experiments, after a visual examination of organ integrity, 15 placentae (623 ± 186 g) collected after caesarean section (*n* = 8) or normal vaginal delivery (n = 7) were used.

### Ex vivo placental perfusion model

The placentae were transported in a thermostatically controlled container (37 °C) and prepared for perfusion within 1 h of delivery in an open double circuit system as previously described [68]. Briefly, an intact peripheral cotyledon was chosen, and the truncal branch of the chorionic artery supplying the cotyledon and its associated vein were catheterized (Microtube Tygon S54-HL 1.02*1.78 mm, Courbevoie, France). The perfusion medium (PM) was Earle’s medium (Euromedex, Souffel Weyersheim, France) supplemented with 2 g/L of BSA (Fraction V, PAA Laboratories, Vélizy-Villacoublay, France), and the foetal flow rate was established at 6 mL/min. After a few minutes, the perfused cotyledon progressively whitened, allowing its isolation and was placed into a thermostatically controlled glass receptacle maintained at 37 °C, with maternal side facing upwards. Two hypodermic cannulas were used to perfuse the maternal side of the placenta with a flow rate established at 12 mL/min. Each placenta was perfused for 30 min with PM alone to flush the blood out of the maternal and foetal circulation and then for 1 h with (*n* = 13) or without (*n* = 2) PM with food-grade (E171) TiO_2_ (15 μg/mL). To ensure proper dispersion, the E171 stock suspension was vigorously agitated using a vortex before addition in the maternal circulation. The perfusion medium was under constant agitation for oxygenation, allowing the homogeneous dispersion of TiO_2_ particles.

During the whole perfusion time (90 min), the foetal and maternal pH values were continuously adjusted throughout the experiments to 7.27 ± 0.05 and 7.41 ± 0.01 (mean ± SD), respectively, and the temperature and flow rates were checked. Antipyrine (Sigma-Aldrich), a reference control substance for passive diffusion and barrier integrity, was added to the maternal reservoir (20 μg/mL) at the beginning of the perfusion (t0), and antipyrine concentrations were measured in foetal exudates collected every 15 min by high performance liquid chromatography coupled with UV detection. All placentae with a maternal to foetal transfer of antipyrine lower than 20% were excluded from the study.

For assessment of the materno-foetal transfer of TiO_2_ particles, repeated samples (2 mL each) from the foetal flow through were collected every 5 min before and after E171 addition on the maternal side and stored at 4 °C after the addition of fungizone (3.5 μL/mL) and penicillin + streptomycin (10 μL/mL) to prevent from bacterial growth. These samples were used for confocal microscopy detection of laser-reflecting particulate matter that reached the foetal side. In addition, pools of foetal exudate (≈50 mL) were collected by 10- or 15-min periods over the whole perfusion time and then stored at − 20 °C for subsequent ICP-MS analysis of the Ti concentration and SEM-EDX detection of the TiO_2_ particles.

At the end of the experiment, the perfused cotyledon was washed for 20 min with PM only to flush out TiO_2_ particles that did not penetrate the tissue. Tissue samples were collected close to the perfusion cannula and taken along the materno-foetal axis as representative areas for particle diffusion from the maternal side to the foetal circuit, while adjacent non-perfused cotyledons were also collected. All these tissue samples were prepared for STEM-EDX observations of particle distribution.

#### ICP-MS analysis of titanium

Placenta biopsies, foetal flow through collected during perfusion and meconium were analysed for Ti concentration with a Thermo Element XR mass spectrometer (ThermoFischer, Bremen, Germany) operated in high resolution mode. Samples were injected with a Seaspray nebulizer in self-aspiration (Glass expansion, Melbourne, Australia) and a quartz double-Scott Peltier-cooled spray chamber. Measurements were performed on isotopes ^47^Ti, ^48^Ti and ^49^Ti, and Ti concentrations were calculated based on the average signals of the three isotopes. The instrument was tuned daily according to the manufacturer’s recommendations, and the Ti spectra were confirmed to be free of interference, particularly calcium interference, and its effect on ^48^Ti was correctly resolved. For foetal exudate, due to the high Ca content (0.49 mM) of the Earle’s medium used for perfusion, only signals on ^47^Ti and ^49^Ti were selected for calculations.

Prior to analysis, placental tissue samples were dry-ashed at 700 °C in a furnace to burn the organic content without damaging the mineral particles. For foetal exudate, each 10-min pool (50 mL) collected during placental perfusion (90 min) was evaporated at 120 °C to near dryness to preconcentrate the sample. Placental tissue ashes and preconcentrated perfusates were digested by the addition of 0.5 mL of HF (40% HF, Suprapur®, Merck) and 10 mL of HNO_3_ (65% HNO_3_, Suprapur®, Merck), evaporated at 120 °C, and suspended in ultrapure water to a final volume of 50 mL. The recovery rates of these procedures were assessed by spiking samples with E171, and yielded 104% for the placental tissues and 85% for the exudates. Digestion blanks and control perfusates (no E171 addition) were used to correct the Ti concentrations from blank contributions.

For meconium, approximately 0.5 mg was sampled and digested in 0.5 mL of HF and 10 mL of HNO_3_ and maintained at room temperature (RT) for 2 days before evaporation at 60 °C. These steps were repeated once or twice until complete digestion. The samples were then diluted in 15 ml of ultrapure water and subsequently diluted in 2% HNO_3_ for analysis. The recovery rates of this procedure were assessed against NIST 1566b and yielded 99.9 ± 10.2% on average (*n* = 2, 1 S.D).

The LOD was calculated as 3 times the standard deviation in the results of a blank sample and the LOQ as 10 times the standard deviation, and the results are shown in mg/kg or ng/mL for each sample.

### Particle detection in the perfusion medium by confocal microscopy

For each perfused placenta, a 35 μL volume droplet of the foetal exudate collected every 5 min from t0 to t90 min of perfusion was placed on 8-well microscopy slides, dried, and mounted in Mowiol medium (glycerol 240 g/L, DABCO 25 g/L, Mowiol 4–88 96 g/L, Tris-HCl 15 g/L) within 24 h after the collect. The slides were then examined under a confocal microscope (Leica SP8) at 488/BP 488–494 nm to detect light scattering (i.e., laser-diffracting) TiO_2_ particles appearing as green fluorescence and at 514/BP 560–660 nm to monitor autofluorescence in the sample, as previously described [7, 8]. Three fields per well were examined, and laser-diffracting (particle) spots were counted using a 63X objective and a magnification factor of 1 pixel to 50 nm. Particles counted in the microscopic fields corresponding to exudate samples from the equilibrium period (from 10 to 30 min) and over the whole experiment with a control placenta perfused with PM alone were considered the background level. To obtain a transfer profile to the foetal circuit of TiO_2_ particles during 1 h after E171 addition in the maternal reservoir, we calculated the background particle level as an average number of particles per observed field, and the value was subtracted from each corresponding value in all placentae perfused with the E171 suspension.

### Preparation of foetal exudate samples for SEM-EDX analysis

To characterize the particles crossing the human placenta, we selected pooled samples from foetal exudate (50 mL) corresponding to the 20–30 min of perfusion after E171 addition in the maternal reservoir based on the particle transfer profile by confocal imaging. Samples were lyophilized to concentrate the particles, and 5 g of powder was suspended in 1 mL of HCl to dissolve organic compounds, sonicated (20 min, 75 W), and centrifuged (10 min, 9503 g) and the supernatant was eliminated. The sample was then washed 5 times as follows: addition of 1 mL of HCl, sonication (1 min, 150 W), centrifugation (10 min, 9503 g) and elimination of the supernatant. The preparation was suspended in 1 mL of HCl, and then, a 7.5 μL droplet was deposited onto a silica wafer by spin-coating according to a method developed by the LNE to perform NP dimensional metrology in complex matrices [69, 70]. Briefly, for good dispersion of the NPs on the silicon substrate while the solvent evaporated, the droplet was spread over the surface of the silica wafer using a low spin speed (1000 rpm, 5 min), and then dried rapidly for 10 s at a fast spin speed (8000 rpm). The samples were stored at RT under a controlled atmosphere until observation.

SEM imaging was performed using a Zeiss Ultra-Plus field emission (FE) microscope equipped with a Gemini optical column. Images were obtained through secondary electrons collected by an InLens detector at a voltage of 3 kV and with a working distance equal to 3 mm. In these working conditions, the provider claimed the resolution of the microscope was 1.7 nm. An EDX detector (Princeton Gamma-Tech Instruments, Princeton, USA) was installed in the SEM chamber for a qualitative elemental analysis of the observed particles. The size distribution of the TiO_2_ particles observed with SEM-EDX was assessed in a semiquantitative way with ImageJ software (NIH, USA), in PM containing 15 μg/mL of E171 and in foetal exudate from 2 placentae.

### Tissue preparation for TEM

Samples from 2 placentae and 2 meconium were fixed in 2% paraformaldehyde-2.5% glutaraldehyde in 0.1 M cacodylate buffer (pH 7.4) for 1 h at 4 °C. For placentae, tissue blocks of 2.5 mm length were excised and immersed in the same fixative overnight at 4 °C. After several rinses in cacodylate buffer, the samples were in 1% OsO_4_ (Osmium (VIII) oxide) for 1 h at 4 °C and rapidly rinsed again. Dehydration was carried out at 4 °C using a graded series of ethanol and acetone. The sections were impregnated with low viscosity epoxy resin (EMS) under a vacuum and polymerized at 60 °C for 48–72 h. Ultrathin sections (50–60 nm, ultra-cut UCT, Leica) were collected on gold− 600 mesh grids and stained for 7 min with 0.5% uranyl acetate in methanol solution before TEM observations. The same protocol was used for meconium samples, except that osmium post-fixation and uranyl staining were not performed.

#### Scanning (S)TEM-EDX analysis, elemental mapping and particle size measurement

TEM-EDX analysis was performed on a JEM 2010 (JEOL, Tokyo, Japan) operating at 120 KV and equipped with a LaB6 cathode and two CCD cameras (Orius 1000 and ES500W) driven by Digital Micrograph software (Gatan-Ametek, Pleasanton, United States). EDX-analysis was performed with a Silicon-drifted Detector (SDD) (Resolution: 125ev Mn_k_) (Oxford-Instruments, Abington, Oxfordshire, England). Elemental mapping was performed on JEM-ARM200F HR-TEM ((JEOL, Tokyo, Japan) with analytical configurations. Elemental maps were acquired at 80 kV in STEM mode with an 8C probe size, a camera length of 8 cm, and a 50 μm condenser aperture with an SDD detector XMax TLE (Resolution: 127ev Mn_k_ 0.7 sr; Oxford-Instruments, Abington, England). Size measurement was determined from bright-field TEM images by using the image processing open-source software ImageJ (NIH, United States).

### Data analysis

The data are presented as the mean ± SD for determination of the TiO_2_ particle diameter in an E171 water suspension and for the Ti dosage in the placentae and the meconium (Table 1, Table 2) or as the mean ± SEM for particle counts and size measurements in the placenta and meconium TEM sections and in the foetal exudate (Figs. 3, and 5).

## Supplementary information

**Additional file 1 Table S1**. ICP-MS analysis of Ti content in foetal exudate collected during control or E171 perfusion. Ti content of foetal exudates from 6 independent E171 perfusion experiments (15 μg/mL) and 1 control perfusion. Samples were collected by time fraction of 10- or (*)15-min during perfusion (first 30 min of 1 h for E5 and E6, total 60 min for E1, E2, E3, E4, and E7). All concentrations are corrected for total blank signals. LOD = 0.23 ng/mL. Typical relative measurement uncertainty was 5% (k = 1). **Fig. S1.** Basal particulate content in term human placenta. (A and B) Representative TEM-Bright Field micrographs showing particulate matter in the placental tissues. Elemental characterization was determined by TEM-EDX analysis. In addition to Carbon and Oxygen, the following elements were found: ①: Tin, Iron, Silicon; ②:Silicon; ③: Tin, Zinc, Iron, Manganese, Phosphorus, Silicon; ④: Silicon; ⑤: Iron, Aluminium, Silicon; ⑥: Silicon, Aluminium; ⑦: Silicon, Aluminium. (C) EDX-Blank spectrum of biological matrix: elements in deconvolution are Uranium (U), Osmium (Os), Gold (Au), Copper (Cu), Chromium (Cr) coming from grids, sample preparation and staining of TEM pieces. **Fig. S2**. Particle diameter distribution in term human placenta. Size measurement of particles recovered per field micrograph on ultrafine sections from 2 placentae collected at term of pregnancy. Dashed line represents the 100 nm limit. **Fig. S3**.Basal particulate content in human meconium. (A to C) TEM-Bright Field micrographs of meconium ultrafine sections showing particles in meconium, and combined TEM-EDX analysis for elemental characterization; ①: Iron (Fe), Silicon (Si), Magnesium (Mg), Calcium (Ca), Aluminium (Al); ②: Titanium (Ti), Aluminium (Al), Silicon (Si). Elements in deconvolution are Gold (Au), Copper (Cu), Chromium (Cr) coming from grids, sample preparation of TEM pieces. **Fig. S4.** Antipyrine rate transfer depending on the placental origin. Foeto-maternal rate transfer of antipyrine during ex vivo perfusions of placentae collected after vaginal delivery (*n* = 5) or caesarean section (*n* = 6). Note that there is no difference in basal permeability depending on the mode of delivery. Data are presented as mean ± SD. **Fig. S5.** Confocal imaging of laser-diffracting particles in perfusion medium with E171, foetal exudate and perfused placenta. (A) Laser-diffracting particles in the E171-containing PM added to maternal side. Scale bar = 50 μm. (B) Foetal exudate collected between 30 and 35 min of E171 perfusion. White arrows indicate laser-diffracting particles. Scale bars = 50 μm. (C) Tissue section of perfused placenta showing laser-diffracting particles spread in the intervillous spaces (ivs) of the maternal side close to the syncytiotrophoblast (sct). White arrows indicate foetal vessels. Scale bar = 100 μm. **Fig. S6.** Complementary EDX analysis of foetal exudates. Purified samples of particles recovered in foetal exudates and showing (A) aluminium (Al) and (B) iron (Fe) elements associated or not with titanium (Ti). Corresponding SEM micrographs of purified particles in upper right panels; Si signal from SEM wafer. **Fig. S7.** Method for size measurement of TIO_2_ particles in the foetal exudate*.* (A) and (B) TiO_2_ particles were identified by an EDX detector coupled to SEM chamber for Ti (green) and O (red) element analysis (upper right panels in A and B), and observed as agglomerates (A) or as isolated particles or small aggregates trapped into the matrix of dried PM after sample preparation, i.e., resulting from the sample deposition onto silicate wafer by spin-coating and evaporation of the solvent [62]. Only the diameters of particles showing Ti + O colocalization (yellow) were measured, here for some particles as examples. Scale bar = 200 nm. **Fig. S8.** Size measurement of TiO_2_ particles in two representative E171-perfused placentae. (A) TEM image reconstruction showing tissue architecture across a placental villus along the materno-foetal axis, i.e., the syncytiotrophoblast microvilli and syncytiotrophoblast cells, the cytotrophoblast cells, then the chorionic mesenchyme composed of the basal lamina supporting trophoblast tissue, and the endothelial cells surrounding foetal capillaries. (B and C) Size distribution of TiO_2_ (EDX-characterized) particles into perfused placenta, (B) trapped into microvilli of the syncytiotrophoblast on the maternal side, and (C) in deeper area until close to foetal vessels. (D) Representative TEM images of perfused TiO_2_ particles (arrowheads) recovered in the intervillous spaces (ivs) close to the syncytiotrophoblast microvilli (D1) and into the placental chorionic mesenchyme surrounding foetal capillaries (D2).

## Data Availability

All relevant data are included in the manuscript and supporting information, and available from the authors upon request.
