# Supplementary material for: Basal Ti level in the human placenta and meconium and evidence of a materno-foetal transfer of food-grade TiO2 nanoparticles in an ex vivo placental perfusion model
Source: Part Fibre Toxicol. 2020 Oct 7;17:51. doi: 10.1186/s12989-020-00381-z (PMC7541303; doi:10.1186/s12989-020-00381-z)
Supplement: Supplementary file 1 — Additional file 1 Table S1. ICP-MS analysis of Ti content in foetal exudate collected during control or E171 perfusion. Ti content of foetal exudates from 6 independent E171 perfusion experiments (15 μg/mL) and 1 control perfusion. Samples were collected by time fraction of 10- or (*)15-min during perfusion (first 30 min of 1 h for E5 and E6, total 60 min for E1, E2, E3, E4, and E7). All concentrations are corrected for total blank signals. LOD = 0.23 ng/mL. Typical relative measurement uncertainty was 5% (k = 1). Fig. S1. Basal particulate content in term human placenta. (A and B) Representative TEM-Bright Field micrographs showing particulate matter in the placental tissues. Elemental characterization was determined by TEM-EDX analysis. In addition to Carbon and Oxygen, the following elements were found: ①: Tin, Iron, Silicon; ②:Silicon; ③: Tin, Zinc, Iron, Manganese, Phosphorus, Silicon; ④: Silicon; ⑤: Iron, Aluminium, Silicon; ⑥: Silicon, Aluminium; ⑦: Silicon, Aluminium. (C) EDX-Blank spectrum of biological matrix: elements in deconvolution are Uranium (U), Osmium (Os), Gold (Au), Copper (Cu), Chromium (Cr) coming from grids, sample preparation and staining of TEM pieces. Fig. S2. Particle diameter distribution in term human placenta. Size measurement of particles recovered per field micrograph on ultrafine sections from 2 placentae collected at term of pregnancy. Dashed line represents the 100 nm limit. Fig. S3.Basal particulate content in human meconium. (A to C) TEM-Bright Field micrographs of meconium ultrafine sections showing particles in meconium, and combined TEM-EDX analysis for elemental characterization; ①: Iron (Fe), Silicon (Si), Magnesium (Mg), Calcium (Ca), Aluminium (Al); ②: Titanium (Ti), Aluminium (Al), Silicon (Si). Elements in deconvolution are Gold (Au), Copper (Cu), Chromium (Cr) coming from grids, sample preparation of TEM pieces. Fig. S4. Antipyrine rate transfer depending on the placental origin. Foeto-maternal rate transfer of antipyri [file 12989_2020_381_MOESM1_ESM.docx]

**Supplemental Material**

**Basal Ti level in the human placenta and meconium and evidence of a materno-foetal transfer of food-grade TiO_2_ nanoparticles in an *ex vivo* placental perfusion model**

Guillard A^1^, Gaultier E^1^, Cartier C^1^, Devoille L^2^, Noireaux J^3^, Chevalier L^4^, Morin M^5^, Grandin F^1^, Lacroix MZ^6^, Coméra C^1^, Cazanave A^1^, de Place A^5^, Gayrard V^1^, Bach V^7^, Chardon K^7^, Bekhti N^8^, Adel-Patient K^8^, Vayssière C^5,9^, Fisicaro P^3^, Feltin N^2^, de la Farge F^1^, Picard-Hagen N^1^, Lamas B^1^, Houdeau E^1*^

**Contents of Supplemental Material**

Supplemental Figure 1

Supplemental Figure 2

Supplemental Figure 3

Supplemental Figure 4

Supplemental Figure 5

Supplemental Figure 6

Supplemental Figure 7

Supplemental Figure 8

Supplemental Table 1


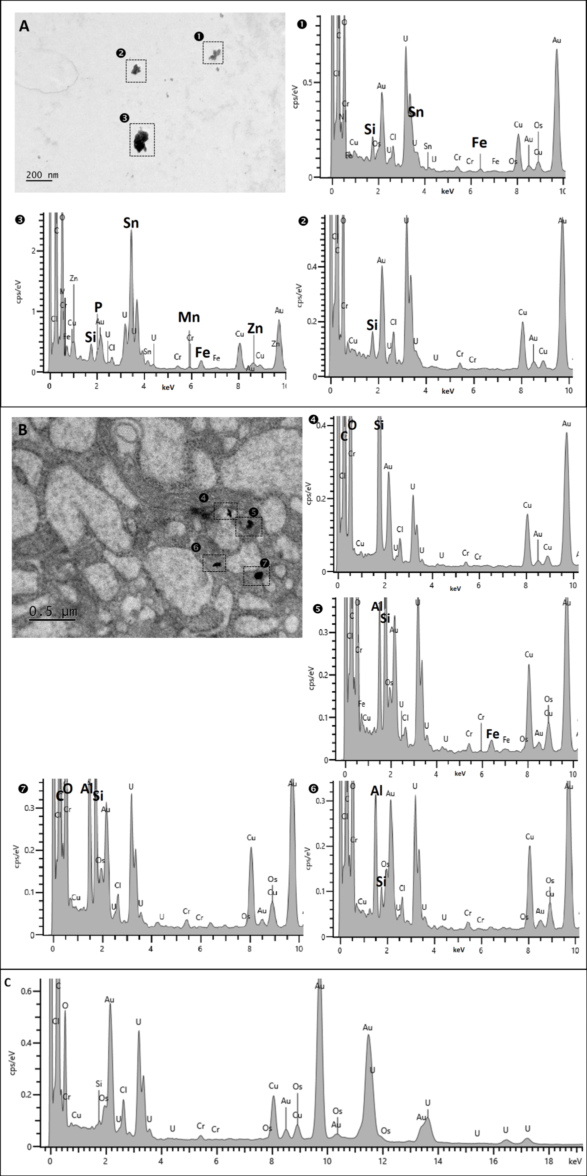


**Figure S1: Basal particulate content in term human placenta.** (A and B) Representative TEM-Bright Field micrographs showing particulate matter in the placental tissues. Elemental characterization was determined by TEM-EDX analysis. In addition to Carbon and Oxygen, the following elements were found: ➊: Tin, Iron, Silicon ; ➋:Silicon; ➌: Tin, Zinc, Iron, Manganese, Phosphorus, Silicon; ➍: Silicon; ➎: Iron, Aluminium, Silicon; ➏: Silicon, Aluminium; ➐: Silicon, Aluminium. (C) EDX-Blank spectrum of biological matrix: elements in deconvolution are Uranium (U), Osmium (Os), Gold (Au), Copper (Cu), Chromium (Cr) coming from grids, sample preparation and staining of TEM pieces.


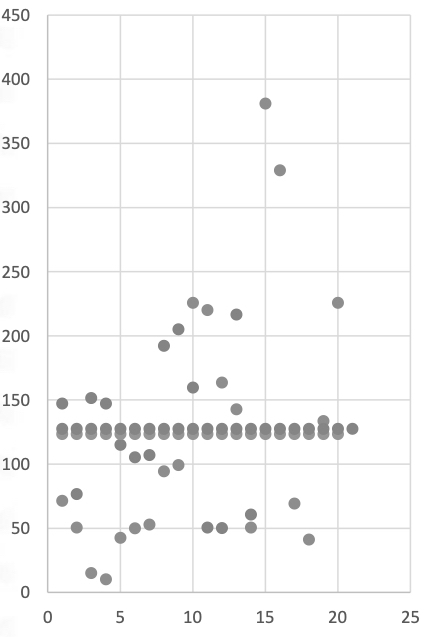


Microscopic (TEM) field

Particle diameter (nm)

**Figure S2: Particle diameter distribution in term human placenta.** Size measurement of particles recovered per field micrograph on ultrafine sections from 2 placentae collected at term of pregnancy. Dashed line represents the 100 nm limit.


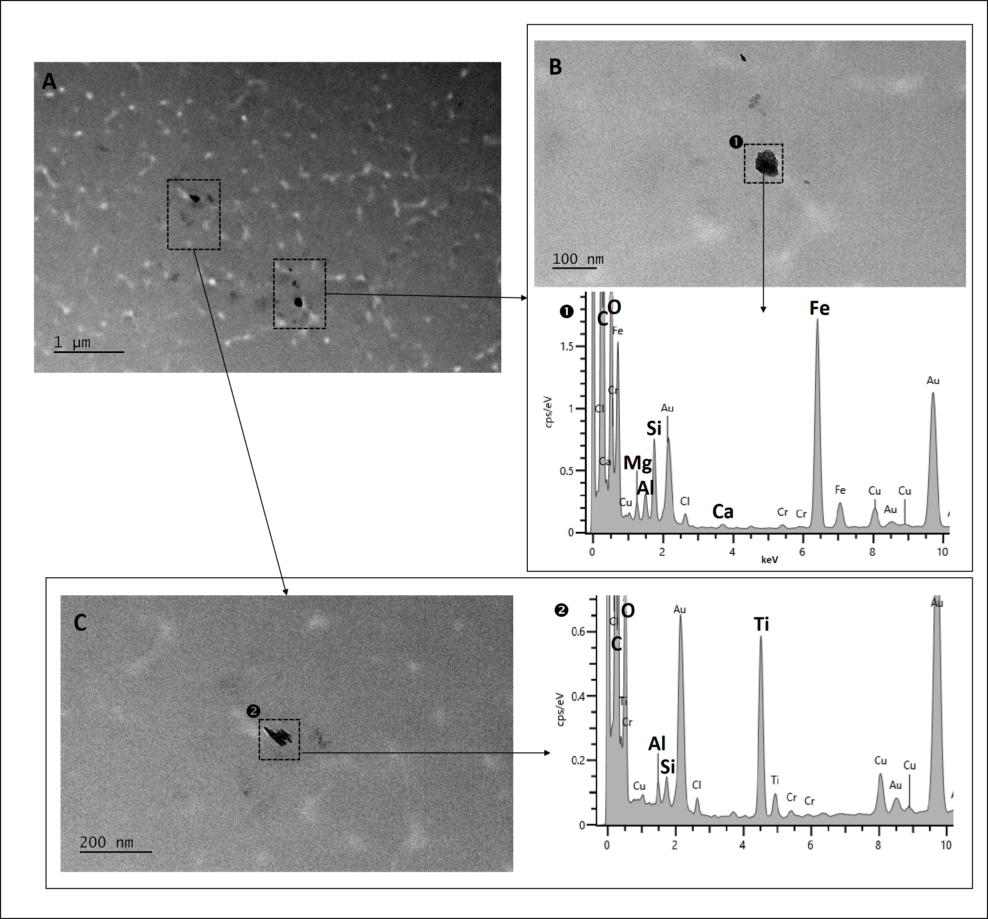


**Figure S3: Basal particulate content in human meconium.** (A to C) TEM-Bright Field micrographs of meconium ultrafine sections showing particles in meconium, and combined TEM-EDX analysis for elemental characterization; ➊: Iron (Fe), Silicon (Si) , Magnesium (Mg), Calcium (Ca), Aluminium (Al); ➋: Titanium (Ti), Aluminium (Al), Silicon (Si). Elements in deconvolution are Gold (Au), Copper (Cu), Chromium (Cr) coming from grids, sample preparation of TEM pieces.

**Figure S4: Antipyrine rate transfer depending on the placenta origin.** Foeto-maternal rate transfer of antipyrine during *ex vivo* perfusions of placentae collected after vaginal delivery (n=5) or caesarean section (n=6). Note that there is no difference in basal permeability depending on the mode of delivery. Data are presented as mean ± SD.


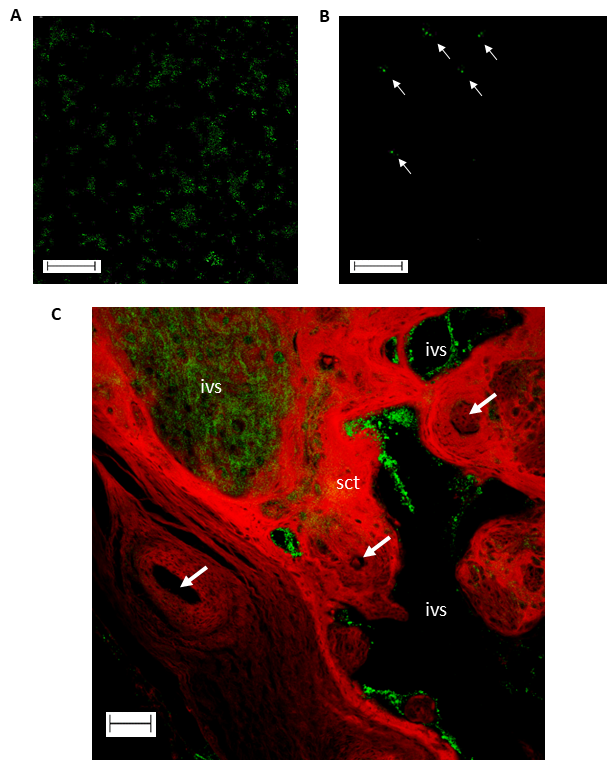


**Figure S5: Confocal imaging of laser-diffracting particles in perfusion medium with E171, foetal exudate and perfused placenta.** (A) Laser-diffracting particles in the E171-containing PM added to maternal side. Scale bar = 50 µm. (B) Foetal exudate collected between 30 and 35 min of E171 perfusion. White arrows indicate laser-diffracting particles. Scale bars = 50 µm. (C) Tissue section of perfused placenta showing laser-diffracting particles spread in the intervillous spaces (ivs) of the maternal side close to the syncytiotrophoblast (sct). White arrows indicate foetal vessels. Scale bar = 100 µm.


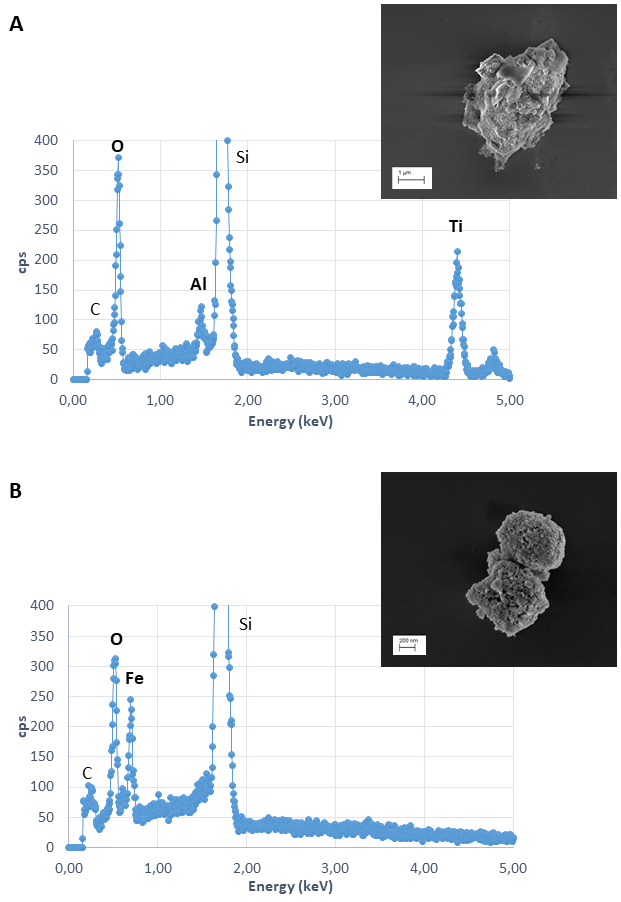


**Figure S6: Complementary EDX analysis of foetal exudates.** Purified samples of particles recovered in foetal exudates and showing (A) aluminium (Al) and (B) iron (Fe) elements associated or not with titanium (Ti). Corresponding SEM micrographs of purified particles in upper right panels; Si signal from SEM wafer.


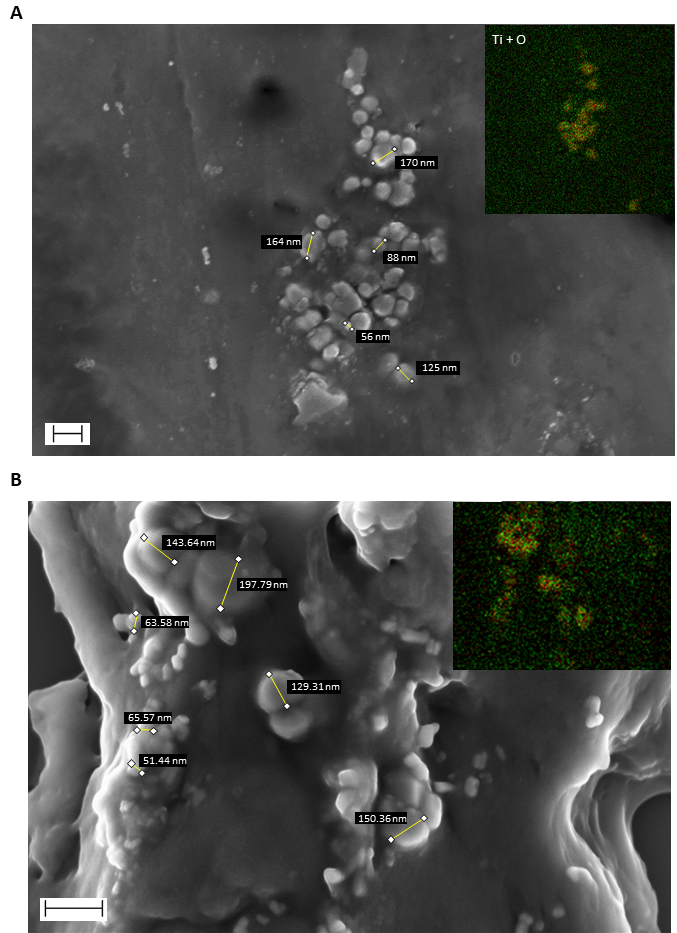


**Figure S7: Method for size measurement of TIO_2_ particles in the foetal exudate***.* (A) and (B) TiO_2_ particles were identified by an EDX detector coupled to SEM chamber for Ti (green) and O (red) element analysis (upper right panels in A and B), and observed as agglomerates (A) or as isolated particles or small aggregates trapped into the matrix of dried PM after sample preparation, i.e., resulting from the sample deposition onto silicate wafer by spin-coating and evaporation of the solvent [62]. Only the diameters of particles showing Ti+O colocalization (yellow) were measured, here for some particles as examples. Scale bar = 200 nm.

**
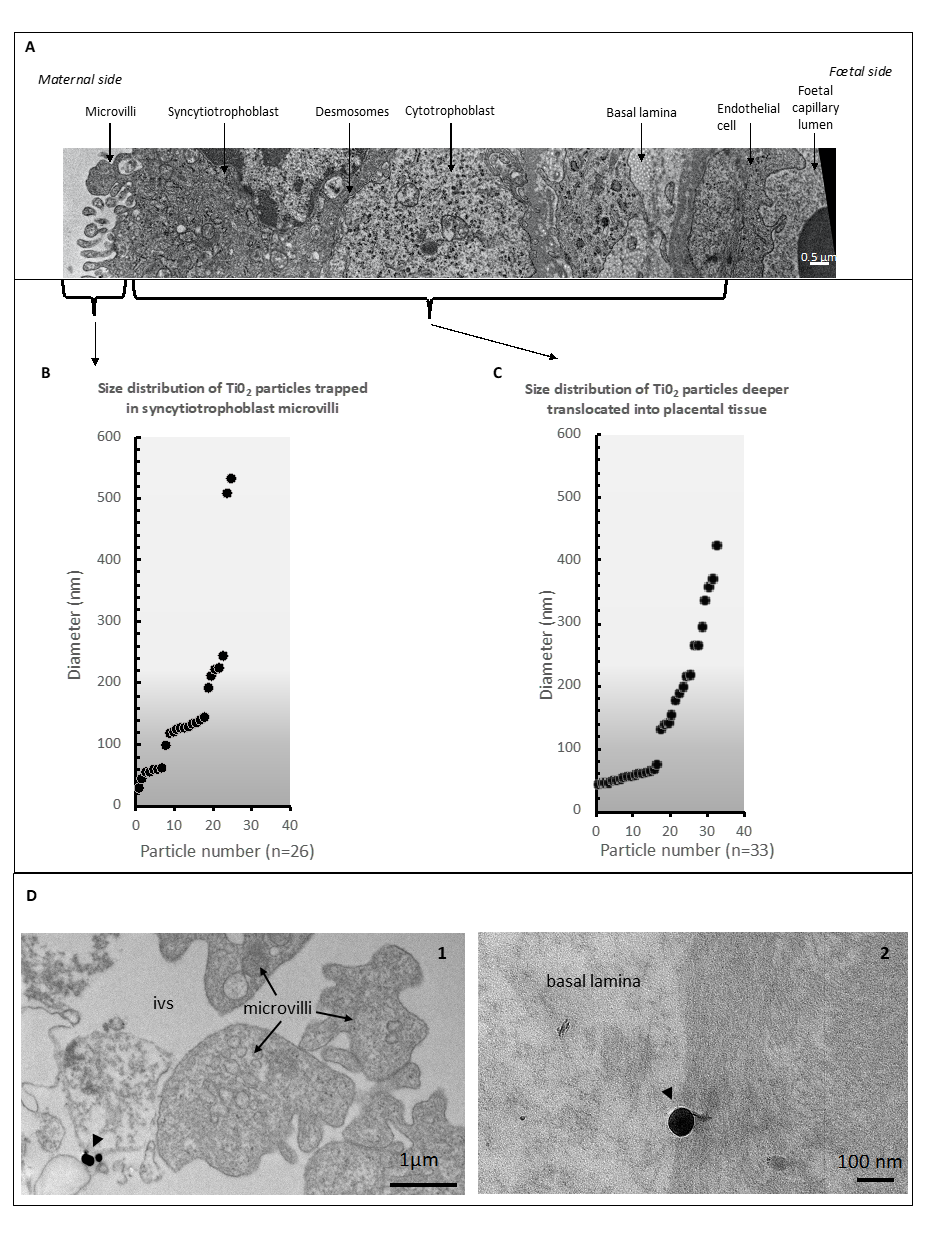
**

**Figure S8: Size measurement of TiO_2_ particles in two representative E171-perfused placentae**. (A) TEM image reconstruction showing tissue architecture across a placental villus along the materno-foetal axis, i.e., the syncytiotrophoblast microvilli and syncytiotrophoblast cells, the cytotrophoblast cells, then the chorionic mesenchyme composed of the basal lamina supporting trophoblast tissue and the endothelial cells surrounding foetal capillaries. (B and C) Size distribution of TiO_2_ (EDX-characterized) particles into perfused placenta, (B) trapped into microvilli of the syncytiotrophoblast on the maternal side (see also Figure 6), and (C) in deeper area until close to foetal vessels. (D) Representative TEM images of perfused TiO_2_ particles (arrowheads) recovered in the intervillous spaces (ivs) close to the syncytiotrophoblast microvilli (D1) and translocated into the placental chorionic mesenchyme surrounding foetal vessels (D2).

| Foetal exudate | Collected fraction | Ti content (ng/mL) |
| --- | --- | --- |
| **E1***  (E171, 15µg/mL) | 1 | *< LOD* |
|  | 2 | *< LOD* |
|  | 3 | *< LOD* |
|  | 4 | *< LOD* |
| **E2***  (E171, 15µg/mL) | 1 | 1,29 |
|  | 2 | 0,41 |
|  | 3 | 1,09 |
|  | 4 | 1,25 |
| **E3*** (E171, 15µg/mL) | 1 | 1 |
|  | 2 | 1,23 |
|  | 3 | 1,58 |
|  | 4 | 1,22 |
| **E4**  (E171, 15µg/mL) | 1 | 1,66 |
|  | 2 | 0,42 |
|  | 3 | *< LOD* |
|  | 4 | *< LOD* |
|  | 5 | *< LOD* |
|  | 6 | *< LOD* |
| **E5**  (E171, 15µg/mL) | 1 | 1,57 |
|  | 2 | 3,46 |
|  | 3 | 2,32 |
| **E6**  (E171, 15µg/mL) | 1 | 1,19 |
|  | 2 | 1,77 |
|  | 3 | 1,11 |
| **E7**  (blank) | 1 | 0,4 |
|  | 2 | 0,56 |
|  | 3 | 0,33 |
|  | 4 | *< LOD* |
|  | 5 | 1,92 |
|  | 6 | 0,61 |

**Table S1: ICP-MS analysis of Ti content in foetal exudate collected during control or E171 perfusion.**

Titanium (Ti) content of foetal exudates (E) from 6 independent E171 perfusion experiments (15 µg/mL) and 1 control (blank) perfusion. Samples were collected by time fraction of 10- or (*)15-min during perfusion (first 30 min of 1 h for E5 and E6, total 60 min for E1, E2, E3, E4, and E7). All concentrations are corrected for total blank signals. LOD = 0.23 ng/mL. Typical relative measurement uncertainty was 5% (k=1).
